# 4-Amino-3-(1-naphthyl­oxymeth­yl)-1*H*-1,2,4-triazole-5(4*H*)-thione

**DOI:** 10.1107/S1600536809051368

**Published:** 2009-12-04

**Authors:** Hoong-Kun Fun, Ching Kheng Quah, A. M. Vijesh, Shridhar Malladi, Arun M. Isloor

**Affiliations:** a X-ray Crystallography Unit, School of Physics, Universiti Sains Malaysia, 11800 USM, Penang, Malaysia; b SeQuent Scientific Limited, No. 120 A & B, Industrial Area, Baikampady, New Mangalore, Karnataka 575 011, India; c Department of Chemistry, National Institute of Technology-Karnataka, Surathkal, Mangalore 575 025, India

## Abstract

In the title compound, C_13_H_12_N_4_OS, the dihedral angle between the triazole and naphthalene ring systems is 67.42 (5)°. In the crystal, adjacent mol­ecules are linked *via* two pairs of inter­molecular N—H⋯S inter­actions, forming *R* ^2^_2_(8) and *R* ^2^_2_(10) ring motifs. Weak C—H⋯S inter­actions generate infinite chains along [001] and the structure is further consolidated by C–H⋯π bonds and aromatic π⋯π stacking inter­actions [distance between the centroids of the triazole rings = 3.2479 (7) Å].

## Related literature

For general background to and the pharmacological activity of triazole derivatives, see: Amir *et al.* (2008 ▶); Sztanke *et al.* (2008 ▶); Kuş *et al.* (2008 ▶); Padmavathi *et al.* (2008 ▶); Isloor *et al.* (2009 ▶). For a related structure, see: Fun *et al.* (2009 ▶). For hydrogen-bond motifs, see: Bernstein *et al.* (1995 ▶). For the preparation, see: Suresh (1992 ▶). For the stability of the temperature controller used for the data collection, see: Cosier & Glazer (1986 ▶).
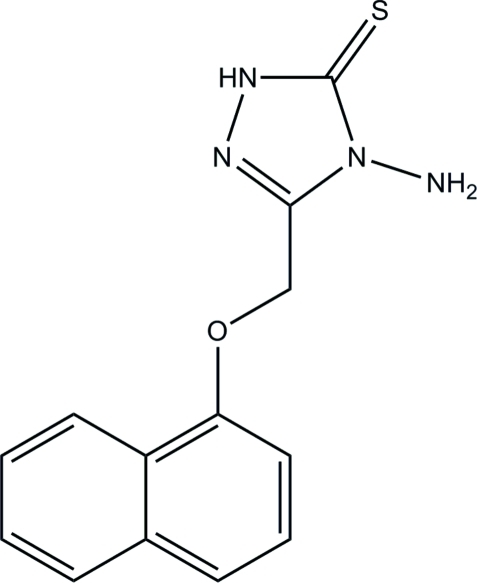


## Experimental

### 

#### Crystal data

C_13_H_12_N_4_OS
*M* *_r_* = 272.33
Monoclinic, 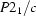

*a* = 7.0023 (1) Å
*b* = 24.0785 (4) Å
*c* = 8.0915 (1) Å
β = 113.404 (1)°
*V* = 1252.02 (3) Å^3^
*Z* = 4
Mo *K*α radiation
μ = 0.26 mm^−1^
*T* = 100 K
0.38 × 0.23 × 0.07 mm

#### Data collection

Bruker SMART APEXII CCD diffractometer
Absorption correction: multi-scan (*SADABS*; Bruker, 2005 ▶) *T*_min_ = 0.908, *T*_max_ = 0.983
24326 measured reflections
5826 independent reflections
4223 reflections with *I* > 2σ(*I*)
*R*_int_ = 0.045

#### Refinement

*R*[*F* ^2^ > 2σ(*F* ^2^)] = 0.045
*wR*(*F* ^2^) = 0.122
*S* = 1.03
5826 reflections
220 parameters
All H-atom parameters refined
Δρ_max_ = 0.51 e Å^−3^
Δρ_min_ = −0.33 e Å^−3^

### 

Data collection: *APEX2* (Bruker, 2005 ▶); cell refinement: *SAINT* (Bruker, 2005 ▶); data reduction: *SAINT*; program(s) used to solve structure: *SHELXTL* (Sheldrick, 2008 ▶); program(s) used to refine structure: *SHELXTL*; molecular graphics: *SHELXTL*; software used to prepare material for publication: *SHELXTL* and *PLATON* (Spek, 2009 ▶).

## Supplementary Material

Crystal structure: contains datablocks global, I. DOI: 10.1107/S1600536809051368/hb5255sup1.cif

Structure factors: contains datablocks I. DOI: 10.1107/S1600536809051368/hb5255Isup2.hkl

Additional supplementary materials:  crystallographic information; 3D view; checkCIF report

## Figures and Tables

**Table 1:** Hydrogen-bond geometry (Å, °)

*D*—H⋯*A*	*D*—H	H⋯*A*	*D*⋯*A*	*D*—H⋯*A*
N1—H1*N*1⋯S1^i^	0.89 (2)	2.39 (2)	3.2857 (11)	176.2 (14)
N4—H1*N*4⋯S1^ii^	0.90 (2)	2.62 (2)	3.5075 (12)	167.3 (19)
C12—H12*A*⋯S1^ii^	0.96 (2)	2.836 (18)	3.5368 (13)	130.3 (12)
C9—H9*A*⋯*Cg*1^iii^	0.964 (17)	2.794 (18)	3.6345 (14)	146.8 (14)

Symmetry codes: (i) 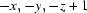; (ii) 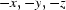; (iii) 
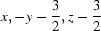
. *Cg*1 is the centroid of C4–C8/C13 ring.

**Fig. 1.**
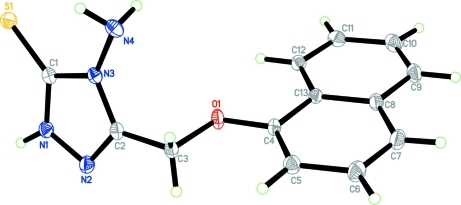
The molecular structure of (I), showing 50% probability displacement ellipsoids for non-H atoms.

**Fig. 2.**
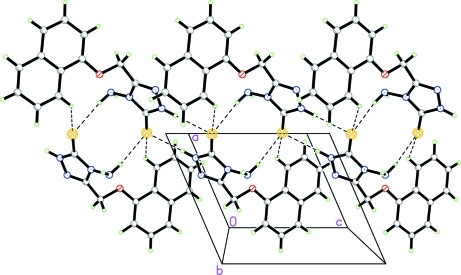
The crystal structure of (I) viewed along the b axis. Intermolecular interactions are shown in dashed lines.

## supplementary crystallographic information

### Comment

1,2,4-triazole and its derivatives were reported to exhibit various pharmacological activities such as antimicrobial, analgesic, anti-inflammatory, anticancer and antioxidant properties (Amir *et al.*, 2008; Sztanke *et al.*., 2008; Kuş *et al.*, 2008; Padmavathi *et al.*, 2008). A few derivatives of triazoles have exhibited antimicrobial activity (Isloor *et al.*, 2009). Some of the present day drugs such as ribavirin (antiviral agent), rizatriptan (anti migraine agent), alprazolam (anxiolytic agent), fluconazole and itraconazole (antifungal agents) are the best examples for potent molecules possessing the triazole nucleus. The amino and mercapto groups of 1,2,4-triazoles serve as readily accessible nucleophilic centers of the preparation of N-bridged heterocycles. Keeping in view of the biological importance, we have synthesized the title compound to study its crystal structure.

The molecular structure of the title compound is shown in Fig. 1. The triazole ring (C1/N1/N2/C2/N3) make an dihedral angle of 67.42 (5)° with naphthalene ring (C4-C13). Short intermolecular distances between the centroids of the triazole rings [3.2479 (7) Å] indicate the existence of π···π interactions. The molecular structure is linked *via* pairs of intermolecular N1—H1N1···S1 and N4—H1N4···S1 interactions, forming *R*^2^_2_ (8) and *R*^2^_2_ (10) ring motifs (Bernstein *et al.*, 1995), respectively. Bond lengths and angles are within normal ranges, and comparable to a closely related structure (Fun *et al.*, 2009). In the crystal packing (Fig. 2), the molecules are linked into infinite one-dimensional chains along the direction [0 0 1] *via* adjacent ring motifs and C12–H12A···S1 interactions (Fig. 2). The crystal strcuture is further consolidated by C–H···π (Table 1) interactions.

### Experimental

2-(1-Naphthyloxy)acetohydrazide (21.6 g, 1.00 mmol) was added slowly to a solution of potassium hydroxide (8.4 g, 1.50 mmol) in ethanol (150 ml). The resulting mixture was stirred well until a clear solution was obtained. Carbon disulphide (11.4 g, 1.50 mmol) was added drop-wise and the contents were stirred vigorously. Further stirring was continued for 24 h. The resulting mixture was diluted with ether (100 ml) and the precipitate formed was collected by filtration, washed with dry ether and dried at 65 /%c under vacuum. It was used for the next step without any purification.

A mixture of the above synthesized potassium dithiocarbazinate (16.5 g, 0.50 mmol), hydrazine hydrate (99 %, 1.00 mmol) and water (2 ml) was heated gently to boil for 30 minutes. Heating was continued until the evacuation of hydrogen sulphide ceased. The reaction mixture was cooled to room temperature, diluted with water (100 ml) and acidified with HCl. The solid mass that separated was collected by filtration, washed with water and dried. Recrystallization was achieved from ethanol. The yield was 9.25 g (68 %), *m. p.* 470-471 K (Suresh, 1992).

### Refinement

All H atoms were located in a difference Fourier map and refined freely.

### Crystal data

**Table:**

C_13_H_12_N_4_OS	*F*(000) = 568
*M**_r_* = 272.33	*D*_x_ = 1.445 Mg m^−^^3^
Monoclinic, *P*2_1_/*c*	Mo *K*α radiation, λ = 0.71073 Å
Hall symbol: -P 2ybc	Cell parameters from 5692 reflections
*a* = 7.0023 (1) Å	θ = 2.9–35.1°
*b* = 24.0785 (4) Å	µ = 0.26 mm^−^^1^
*c* = 8.0915 (1) Å	*T* = 100 K
β = 113.404 (1)°	Plate, yellow
*V* = 1252.02 (3) Å^3^	0.38 × 0.23 × 0.07 mm
*Z* = 4	

### Data collection

**Table:**

Bruker SMART APEXII CCD diffractometer	5826 independent reflections
Radiation source: fine-focus sealed tube	4223 reflections with *I* > 2σ(*I*)
graphite	*R*_int_ = 0.045
φ and ω scans	θ_max_ = 35.8°, θ_min_ = 2.9°
Absorption correction: multi-scan (*SADABS*; Bruker, 2005)	*h* = −11→11
*T*_min_ = 0.908, *T*_max_ = 0.983	*k* = −39→38
24326 measured reflections	*l* = −13→13

### Refinement

**Table:**

Refinement on *F*^2^	Primary atom site location: structure-invariant direct methods
Least-squares matrix: full	Secondary atom site location: difference Fourier map
*R*[*F*^2^ > 2σ(*F*^2^)] = 0.045	Hydrogen site location: inferred from neighbouring sites
*wR*(*F*^2^) = 0.122	All H-atom parameters refined
*S* = 1.03	*w* = 1/[σ^2^(*F*_o_^2^) + (0.0592*P*)^2^ + 0.214*P*] where *P* = (*F*_o_^2^ + 2*F*_c_^2^)/3
5826 reflections	(Δ/σ)_max_ < 0.001
220 parameters	Δρ_max_ = 0.51 e Å^−^^3^
0 restraints	Δρ_min_ = −0.33 e Å^−^^3^

### Special details

**Table:**

Experimental. The crystal was placed in the cold stream of an Oxford Cyrosystems Cobra open-flow nitrogen cryostat (Cosier & Glazer, 1986) operating at 100.0 (1) K.
Geometry. All esds (except the esd in the dihedral angle between two l.s. planes) are estimated using the full covariance matrix. The cell esds are taken into account individually in the estimation of esds in distances, angles and torsion angles; correlations between esds in cell parameters are only used when they are defined by crystal symmetry. An approximate (isotropic) treatment of cell esds is used for estimating esds involving l.s. planes.
Refinement. Refinement of F^2^ against ALL reflections. The weighted R-factor wR and goodness of fit S are based on F^2^, conventional R-factors R are based on F, with F set to zero for negative F^2^. The threshold expression of F^2^ > 2sigma(F^2^) is used only for calculating R-factors(gt) etc. and is not relevant to the choice of reflections for refinement. R-factors based on F^2^ are statistically about twice as large as those based on F, and R- factors based on ALL data will be even larger.

### Fractional atomic coordinates and isotropic or equivalent isotropic displacement parameters (Å^2^)

**Table:**

	*x*	*y*	*z*	*U*_iso_*/*U*_eq_	
S1	0.00629 (5)	0.044619 (13)	0.25791 (4)	0.01810 (8)	
O1	0.52129 (13)	−0.11362 (4)	0.14188 (11)	0.01666 (16)	
N1	0.22651 (15)	−0.04341 (4)	0.46060 (13)	0.01481 (17)	
N2	0.38889 (16)	−0.07854 (4)	0.47914 (13)	0.01636 (18)	
N3	0.33179 (15)	−0.01814 (4)	0.25913 (12)	0.01411 (17)	
N4	0.37675 (18)	0.01243 (5)	0.13172 (14)	0.0193 (2)	
C1	0.18602 (17)	−0.00623 (5)	0.32721 (14)	0.01419 (19)	
C2	0.44981 (17)	−0.06189 (5)	0.35421 (15)	0.01418 (19)	
C3	0.61978 (18)	−0.08762 (5)	0.31417 (15)	0.0162 (2)	
C4	0.65096 (17)	−0.13872 (5)	0.07391 (14)	0.01338 (19)	
C5	0.86351 (18)	−0.14187 (5)	0.16169 (16)	0.0165 (2)	
C6	0.98108 (19)	−0.16981 (5)	0.07908 (17)	0.0195 (2)	
C7	0.88492 (19)	−0.19351 (5)	−0.08729 (17)	0.0187 (2)	
C8	0.66644 (18)	−0.19040 (5)	−0.18076 (15)	0.01484 (19)	
C9	0.5615 (2)	−0.21540 (5)	−0.35253 (16)	0.0185 (2)	
C10	0.3499 (2)	−0.21323 (5)	−0.43817 (16)	0.0199 (2)	
C11	0.2304 (2)	−0.18580 (5)	−0.35758 (16)	0.0178 (2)	
C12	0.32576 (18)	−0.16061 (5)	−0.19258 (15)	0.01452 (19)	
C13	0.54499 (17)	−0.16261 (4)	−0.10061 (14)	0.01255 (18)	
H3A	0.693 (2)	−0.1148 (6)	0.405 (2)	0.013 (3)*	
H3B	0.715 (2)	−0.0602 (6)	0.310 (2)	0.015 (4)*	
H5A	0.940 (3)	−0.1254 (7)	0.279 (2)	0.022 (4)*	
H6A	1.131 (3)	−0.1723 (7)	0.134 (2)	0.029 (4)*	
H7A	0.962 (3)	−0.2110 (7)	−0.143 (2)	0.026 (4)*	
H9A	0.647 (3)	−0.2335 (7)	−0.404 (2)	0.031 (5)*	
H10A	0.288 (3)	−0.2318 (7)	−0.550 (2)	0.028 (4)*	
H11A	0.080 (3)	−0.1865 (7)	−0.414 (2)	0.023 (4)*	
H12A	0.240 (3)	−0.1418 (7)	−0.142 (2)	0.022 (4)*	
H1N1	0.162 (3)	−0.0455 (7)	0.536 (3)	0.031 (5)*	
H1N4	0.284 (3)	0.0023 (8)	0.022 (3)	0.035 (5)*	
H2N4	0.354 (3)	0.0475 (8)	0.153 (3)	0.035 (5)*	

### Atomic displacement parameters (Å^2^)

**Table:**

	*U*^11^	*U*^22^	*U*^33^	*U*^12^	*U*^13^	*U*^23^
S1	0.01841 (14)	0.02112 (15)	0.01629 (13)	0.00540 (10)	0.00852 (11)	0.00193 (10)
O1	0.0150 (4)	0.0207 (4)	0.0148 (3)	0.0010 (3)	0.0065 (3)	−0.0072 (3)
N1	0.0161 (4)	0.0159 (4)	0.0151 (4)	0.0006 (3)	0.0090 (4)	−0.0001 (3)
N2	0.0187 (4)	0.0159 (4)	0.0170 (4)	0.0015 (4)	0.0098 (4)	−0.0006 (3)
N3	0.0157 (4)	0.0156 (4)	0.0129 (4)	0.0004 (3)	0.0077 (3)	−0.0001 (3)
N4	0.0248 (5)	0.0216 (5)	0.0153 (4)	0.0013 (4)	0.0122 (4)	0.0031 (4)
C1	0.0144 (5)	0.0157 (5)	0.0133 (4)	−0.0014 (4)	0.0064 (4)	−0.0022 (3)
C2	0.0151 (5)	0.0143 (5)	0.0138 (4)	−0.0005 (4)	0.0064 (4)	−0.0029 (3)
C3	0.0157 (5)	0.0191 (5)	0.0135 (4)	0.0012 (4)	0.0056 (4)	−0.0044 (4)
C4	0.0141 (4)	0.0139 (5)	0.0140 (4)	0.0013 (3)	0.0076 (4)	−0.0015 (3)
C5	0.0148 (5)	0.0188 (5)	0.0156 (5)	0.0000 (4)	0.0057 (4)	−0.0023 (4)
C6	0.0145 (5)	0.0233 (6)	0.0218 (5)	0.0027 (4)	0.0085 (4)	−0.0006 (4)
C7	0.0187 (5)	0.0193 (5)	0.0218 (5)	0.0035 (4)	0.0118 (5)	−0.0007 (4)
C8	0.0187 (5)	0.0130 (5)	0.0159 (4)	0.0008 (4)	0.0100 (4)	0.0001 (4)
C9	0.0259 (6)	0.0157 (5)	0.0172 (5)	−0.0006 (4)	0.0120 (5)	−0.0034 (4)
C10	0.0261 (6)	0.0174 (5)	0.0153 (5)	−0.0032 (4)	0.0072 (5)	−0.0039 (4)
C11	0.0185 (5)	0.0177 (5)	0.0159 (5)	−0.0026 (4)	0.0054 (4)	0.0000 (4)
C12	0.0156 (5)	0.0137 (5)	0.0148 (4)	−0.0003 (4)	0.0066 (4)	−0.0003 (4)
C13	0.0145 (5)	0.0111 (4)	0.0129 (4)	0.0004 (3)	0.0065 (4)	−0.0003 (3)

### Geometric parameters (Å, °)

**Table:**

S1—C1	1.6842 (12)	C5—C6	1.4192 (16)
O1—C4	1.3743 (12)	C5—H5A	0.971 (17)
O1—C3	1.4308 (13)	C6—C7	1.3678 (18)
N1—C1	1.3431 (14)	C6—H6A	0.964 (18)
N1—N2	1.3768 (13)	C7—C8	1.4138 (17)
N1—H1N1	0.894 (19)	C7—H7A	0.931 (17)
N2—C2	1.3066 (14)	C8—C9	1.4223 (16)
N3—C1	1.3691 (13)	C8—C13	1.4246 (15)
N3—C2	1.3710 (15)	C9—C10	1.3648 (19)
N3—N4	1.4005 (13)	C9—H9A	0.964 (17)
N4—H1N4	0.90 (2)	C10—C11	1.4123 (17)
N4—H2N4	0.888 (19)	C10—H10A	0.947 (18)
C2—C3	1.4872 (15)	C11—C12	1.3740 (16)
C3—H3A	0.966 (15)	C11—H11A	0.967 (17)
C3—H3B	0.948 (15)	C12—C13	1.4160 (16)
C4—C5	1.3732 (16)	C12—H12A	0.960 (16)
C4—C13	1.4294 (15)		
			
C4—O1—C3	116.29 (9)	C4—C5—H5A	123.0 (10)
C1—N1—N2	113.50 (9)	C6—C5—H5A	117.3 (10)
C1—N1—H1N1	125.7 (12)	C7—C6—C5	120.69 (11)
N2—N1—H1N1	120.7 (12)	C7—C6—H6A	116.6 (10)
C2—N2—N1	103.67 (9)	C5—C6—H6A	122.6 (10)
C1—N3—C2	108.34 (9)	C6—C7—C8	120.62 (10)
C1—N3—N4	127.42 (10)	C6—C7—H7A	121.0 (11)
C2—N3—N4	123.73 (9)	C8—C7—H7A	118.4 (11)
N3—N4—H1N4	107.3 (12)	C7—C8—C9	121.97 (10)
N3—N4—H2N4	104.3 (12)	C7—C8—C13	119.74 (10)
H1N4—N4—H2N4	109.4 (18)	C9—C8—C13	118.27 (10)
N1—C1—N3	103.33 (9)	C10—C9—C8	121.01 (10)
N1—C1—S1	130.05 (8)	C10—C9—H9A	122.4 (11)
N3—C1—S1	126.60 (9)	C8—C9—H9A	116.6 (11)
N2—C2—N3	111.15 (10)	C9—C10—C11	120.41 (11)
N2—C2—C3	125.18 (11)	C9—C10—H10A	117.4 (10)
N3—C2—C3	123.64 (10)	C11—C10—H10A	122.2 (10)
O1—C3—C2	106.07 (9)	C12—C11—C10	120.45 (11)
O1—C3—H3A	110.6 (9)	C12—C11—H11A	119.1 (10)
C2—C3—H3A	109.9 (8)	C10—C11—H11A	120.4 (10)
O1—C3—H3B	110.0 (9)	C11—C12—C13	120.17 (10)
C2—C3—H3B	110.6 (9)	C11—C12—H12A	118.5 (10)
H3A—C3—H3B	109.6 (13)	C13—C12—H12A	121.4 (10)
C5—C4—O1	124.75 (10)	C12—C13—C8	119.68 (10)
C5—C4—C13	121.29 (9)	C12—C13—C4	122.31 (9)
O1—C4—C13	113.96 (9)	C8—C13—C4	117.98 (10)
C4—C5—C6	119.66 (11)		
			
C1—N1—N2—C2	0.50 (13)	C4—C5—C6—C7	0.18 (19)
N2—N1—C1—N3	−0.61 (12)	C5—C6—C7—C8	0.32 (19)
N2—N1—C1—S1	−179.18 (9)	C6—C7—C8—C9	−178.92 (12)
C2—N3—C1—N1	0.48 (12)	C6—C7—C8—C13	−0.36 (18)
N4—N3—C1—N1	−171.51 (10)	C7—C8—C9—C10	178.15 (11)
C2—N3—C1—S1	179.11 (9)	C13—C8—C9—C10	−0.43 (17)
N4—N3—C1—S1	7.12 (17)	C8—C9—C10—C11	0.18 (18)
N1—N2—C2—N3	−0.17 (12)	C9—C10—C11—C12	0.48 (18)
N1—N2—C2—C3	−178.26 (11)	C10—C11—C12—C13	−0.87 (17)
C1—N3—C2—N2	−0.20 (13)	C11—C12—C13—C8	0.61 (16)
N4—N3—C2—N2	172.15 (10)	C11—C12—C13—C4	−177.82 (11)
C1—N3—C2—C3	177.92 (10)	C7—C8—C13—C12	−178.58 (11)
N4—N3—C2—C3	−9.73 (17)	C9—C8—C13—C12	0.03 (16)
C4—O1—C3—C2	177.76 (9)	C7—C8—C13—C4	−0.08 (16)
N2—C2—C3—O1	110.95 (12)	C9—C8—C13—C4	178.53 (10)
N3—C2—C3—O1	−66.91 (14)	C5—C4—C13—C12	179.03 (11)
C3—O1—C4—C5	2.21 (16)	O1—C4—C13—C12	−0.50 (15)
C3—O1—C4—C13	−178.28 (9)	C5—C4—C13—C8	0.57 (16)
O1—C4—C5—C6	178.85 (11)	O1—C4—C13—C8	−178.96 (9)
C13—C4—C5—C6	−0.63 (17)		

### Hydrogen-bond geometry (Å, °)

**Table:**

*D*—H···*A*	*D*—H	H···*A*	*D*···*A*	*D*—H···*A*
N1—H1N1···S1^i^	0.89 (2)	2.39 (2)	3.2857 (11)	176.2 (14)
N4—H1N4···S1^ii^	0.90 (2)	2.62 (2)	3.5075 (12)	167.3 (19)
C12—H12A···S1^ii^	0.96 (2)	2.836 (18)	3.5368 (13)	130.3 (12)
C9—H9A···Cg1^iii^	0.964 (17)	2.794 (18)	3.6345 (14)	146.8 (14)

Symmetry codes: (i) −*x*, −*y*, −*z*+1; (ii) −*x*, −*y*, −*z*; (iii) *x*, −*y*−3/2, *z*−3/2.

## References

[bb1] Amir, M., Kumar, H. & Javed, S. A. (2008). *Eur. J. Med. Chem.* **43**, 2056–2066. 10.1016/j.ejmech.2007.09.025 18023930

[bb2] Bernstein, J., Davis, R. E., Shimoni, L. & Chamg, N.-L. (1995). *Angew. Chem. Int. Ed. Engl.* **34**, 1555–1573.

[bb3] Bruker (2005). *APEX2*, *SAINT* and *SADABS* Bruker AXS Inc., Madison, Wisconsin, USA.

[bb4] Cosier, J. & Glazer, A. M. (1986). *J. Appl. Cryst.* **19**, 105–107.

[bb5] Fun, H.-K., Liew, W.-C., Vijesh, A. M., Padaki, M. & Isloor, A. M. (2009). *Acta Cryst.* E**65**, o1910–o1911. 10.1107/S1600536809027275 PMC2977493 21583598

[bb6] Isloor, A. M., Kalluraya, B. & Shetty, P. (2009). *Eur. J. Med. Chem.* **44**, 3784–3787. 10.1016/j.ejmech.2009.04.038 19464087

[bb7] Kuş, C., Ayhan Kılcıgil, G., Zbey, O. S., Kaynak, F. B., Kaya, M., Oban, C. T. & Can-Eke, B. (2008). *Bioorg. Med. Chem.* **16**, 4294–4303. 10.1016/j.bmc.2008.02.077 18337107

[bb8] Padmavathi, V., Thriveni, P., Reddy, G. S. & Deepti, D. (2008). *Eur. J. Med. Chem.* **43**, 917–924. 10.1016/j.ejmech.2007.06.011 17692433

[bb9] Sheldrick, G. M. (2008). *Acta Cryst.* A**64**, 112–122. 10.1107/S0108767307043930 18156677

[bb10] Spek, A. L. (2009). *Acta Cryst.* D**65**, 148–155. 10.1107/S090744490804362X PMC2631630 19171970

[bb12] Suresh K. V. (1992). M Phil dissertation, Mangalore University, India.

[bb11] Sztanke, K., Tuzimski, T., Rzymowska, J., Pasternak, K. & Kandefer-Szerszeń, M. (2008). *Eur. J. Med. Chem.* **43**, 404–419. 10.1016/j.ejmech.2007.03.033 17531354

